# Diffusion tensor imaging correlates with lesion volume in cerebral hemisphere infarctions

**DOI:** 10.1186/1471-2342-10-21

**Published:** 2010-09-17

**Authors:** Maija E Rossi, Eeva Jason, Silvia Marchesotti, Prasun Dastidar, Jyrki Ollikainen, Seppo Soimakallio

**Affiliations:** 1 Medical Imaging Centre, Department of Radiology, Tampere University Hospital, Tampere, Finland; 2 Tampere Medical School, Tampere, Finland; 3 University of Genoa, Genoa, Italy; 4 Clinics of Neurology and Rehabilitation, Tampere University Hospital, Tampere, Finland

## Abstract

**Background:**

Both a large lesion volume and abnormalities in diffusion tensor imaging are independently associated with a poor prognosis after cerebral infarctions. Therefore, we assume that they are associated. This study assessed the associations between lesion volumes and diffusion tensor imaging in patients with a right-sided cerebral infarction.

**Methods:**

The lesion volumes of 33 patients (age 65.9 ± 8.7, 26 males and 7 females) were imaged using computed tomography (CT) in the acute phase (within 3-4 hours) and magnetic resonance imaging (MRI) in the chronic phase (follow-up at 12 months, with a range of 8-27 months). The chronic-phase fractional anisotropy (FA) and mean diffusivity (MD) values were measured at the site of the infarct and selected white matter tracts. Neurological tests in both the acute and chronic phases, and DTI lateralization were assessed with the Wilcoxon signed-rank test. The effects of thrombolytic therapy (n = 10) were assessed with the Mann-Whitney U test. The correlations between the measured parameters were analysed with Spearman's rho correlation. Bonferroni post-hoc correction was used to compensate for the familywise error rate in multiple comparisons.

**Results:**

Several MD values in the right hemisphere correlated positively and FA values negatively with the lesion volumes. These correlations included both lesion area and healthy tissue. The results of the mini-mental state examination and the National Institutes of Health Stroke Scale also correlated with the lesion volume.

**Conclusions:**

A larger infarct volume is associated with more pronounced tissue modifications in the chronic stage as observed with the MD and FA alterations.

## Background

Patients with a right-sided cerebral infarction and attention deficits are known to have a worse functional outcome in comparison to patients with a left-sided infarction [1,2]. However, the anatomical correlate of an attention deficit is still uncertain and associated with both the middle cerebral artery (MCA) and the posterior cerebral artery (PCA) [3]. Patients with cerebral artery infarctions and attention deficits have disconnections both between and within hemispheres [3,4].

Diffusion tensor imaging (DTI) of stroke patients is an important imaging method in radiological research, representing a useful tool when conventional imaging methods are insufficient. DTI parameters have been reported to show early signs of Wallerian degeneration [5] and to correlate with the results of neurological tests [6]. The fractional anisotropy (FA) and mean diffusivity (MD) changes both in the infarcted area and in seemingly healthy brain areas are now obvious, though not yet fully understood [6-8].

Volumetric estimations of cerebral infarctions and their prognostic value have been studied widely. Lesion volumes have been shown to correlate with clinical outcomes [9-11]. Both magnetic resonance imaging (MRI) and computed tomography (CT) have been used in the evaluation of lesion volumes. Pantano *et al. *[11] reported that early increases in lesion volumes observed on CT during the first week are related to more severe neurological deficits and lower degrees of neurological improvement. Both acute and chronic lesion volumes in diffusion-weighted imaging (DWI) are reported to correlate with the clinical severity as measured by the National Institutes of Health Stroke Scale (NIHSS) [9,10]. In addition, a change in the lesion volume correlated with a change in the clinical scores [10]. Although MRI has proven to be superior to CT in evaluating acute strokes [12,13], CT is still the most commonly used imaging modality in the assessment of stroke patients.

Both DTI and lesion volumes have been individually investigated and show promise. Therefore, we expected them to associate with one another. We performed CTs in the early phase of ischemia and MRIs, including DTI, in the chronic phase in patients with right-sided cerebral infarctions that are known to have worse outcomes than left-sided infarctions [1,2]. Our study is the first one describing the associations between lesion volume and the DTI parameters FA and MD. The infarct volumes will be also shown to correlate with neurological deficits.

## Methods

### Patients

We studied 41 consecutive patients diagnosed with right cerebral hemisphere infarctions and suspected for attention deficit. The patients were treated in accordance with standard procedures in the Acute Neurology Ward of Tampere University Hospital between July 2005 and June 2008. Written informed consent was obtained from all patients. The study was approved by the Ethical Committee of Tampere University Hospital.

### Inclusion and exclusion criteria

The patients included in the study had had their first infarction, were right-handed and were less than 80 years old. They had also been capable of living independently before the infarction. Patients were excluded from the study if they had previous neurological or psychiatric disorders, a history of drug abuse, previous lesions in the brain found on acute CT, remarkable brain atrophy in comparison to the patient's age, severe hearing or primary visual impairments or a major decline in consciousness. Moreover, patients without signs of an infarction on imaging in the chronic phase were excluded from the study.

### Time frame of the study

A CT was performed on all patients in the acute phase in less than 3-4 hours of the onset of symptoms, including perfusion CT and angiography. Ten patients then received thrombolytic therapy [14], and another CT was performed within 24 hours. For this study, only the conventional CT obtained prior to the administration of thrombolytics was used for the volumetric analysis. A follow-up MRI was performed in the chronic phase at an average of 13 months (range 8-27 months, interquartile range (IQR) 12 to 21 months) after the infarction. Patients underwent rehabilitation in accordance with the routine protocol of the acute unit.

Neurological tests were conducted in the acute phase (within ten days of the infarction) and in the chronic phase six months after the infarction. The same neurological tests were performed each time and included the Barthel Index (BI), the NIHSS, the Rankin Scale (RS) and the mini-mental state examination (MMSE).

### Imaging Sequences

As an ongoing project in the Stroke Unit of our university hospital, the patients were routinely imaged 3-4 hours from the onset using conventional CT, CT angiography, and perfusion CT. The conventional CT is used to rule out haemorrhage, visible acute infarctions, and significant microangiopathy. CT angiography is performed to image acute carotid or vertebral artery dissections or blocks. Perfusion CT is performed to rule out perfusion disturbances in the suspected ischemic area. The CT scans were obtained using either a Philips Brilliance64 (Best, Netherlands) or GE Lightspeed RT16 (Wisconsin, USA), depending on the availability of the scanner. The imaging parameters were 120 kVp on both scanners; and 430 mAs on Philips and 320 mAs on GE. The in-plane resolution was 0.45*0.45 mm^2^, and the slice thickness was 5 mm.

The MRIs were performed with a 1.5-T MRI whole-body scanner (Magnetom Avanto SQ, Siemens Medical Solutions, Erlangen, Germany) equipped with gradient hardware allowing gradients up to 40 mT/m. A conventional 12-channel head matrix coil was used. The whole MRI protocol included sagittal *T *_1_-weighted spin echo (SE), 3D *T *_2_-weighted SPACE sequences, axial *T *_1 _SE, *T *_2_-weighted fluid-attenuated inversion recovery (FLAIR), *T *_2_-weighted and diffusion-weighted sequences. In this study, we used diffusion-weighted and *T *_2_-weighted FLAIR sequences.

The diffusion protocol consisted of a single-shot diffusion weighted echo-planar imaging (EPI) sequence. Typical acquisition parameters were as follows: repetition time (TR): 3500 ms, echo time (TE): 96 ms, slice thickness: 5 mm, interslice gap: 1.5 mm, field of view (FOV): 23 × 23 cm, matrix: 128 × 128 (in-plane resolution = 1.8*1.8 mm^2^), b value: 1000 and 0 s/mm^2^, number of excitations = 3, and 12 gradient encoding directions. For the volumetric analysis, we used a *T *_2_-weighted FLAIR sequence with the following parameters: TR 8500 ms, TE 100 ms, inversion time 2500 ms, slice thickness 5 mm, interslice gap 1.5 mm, FOV 23 × 23 cm and matrix 256 × 256 interpolated to 512 × 512 (in-plane resolution = 0.45*0.45 mm^2^). In both sequences, the only slight exceptions to these parameters were made in the FOV due to the size of the patient and consequently, the in-plane resolution.

### DTI Analysis

**Figure 1:**
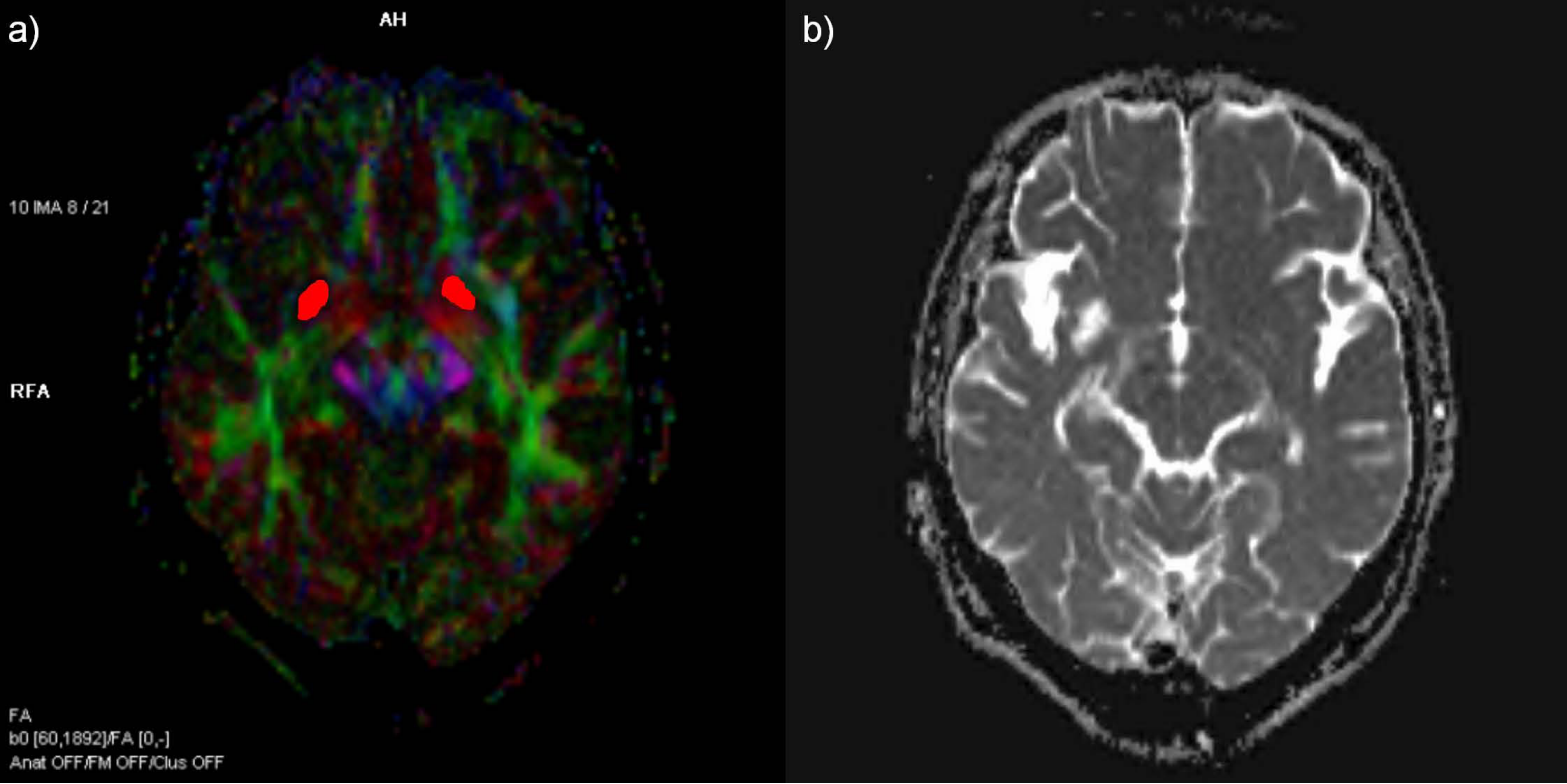
**Infarction lesion**. The right-hemispheric lesion area and the corresponding contralateral area (a) in diffusion images with regions of interest marked in red. The corresponding lesion is shown in an apparent diffusion coefficient map (b). The high-signal infarction area is situated at the junction of the external capsule and the caudate nucleus.

**Figure 2:**
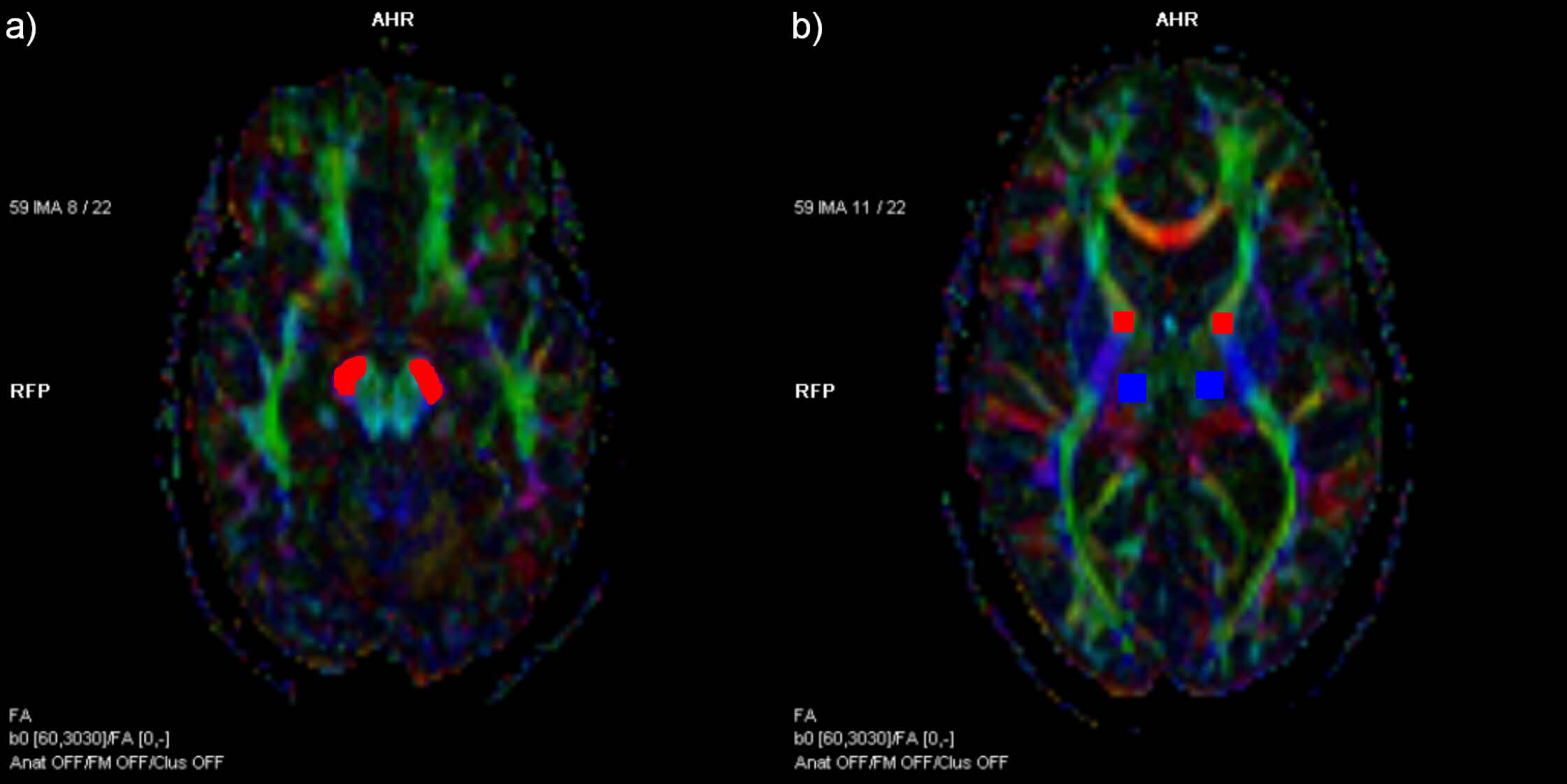
**Examples of region of interest setting**. (a) Region of interest setting in the cerebral peduncle (red); (b) the knee of the internal capsule (red) and the thalamus (blue).

The DTI data was analysed with commercial software (Siemens Syngo VE26A, Neuro 3D, Siemens, Erlangen, Germany) on an offline workstation. We measured the DTI parameters:

{MD=​(λ1+λ2+λ3)/3FA=12*(λ1−λ2)2+(λ2−λ3)2+(λ3−λ1)2λ12+λ22+λ32

where λ_n _is the diffusivity along the n^th ^axis, and [MD] = (× 10^-3 ^mm^2^s^-1^) [15]. The FA and MD values of all patients were carefully measured by the same reader (E.J.). The regions of interest (ROIs) were manually defined. The ROIs were symmetrically placed on axial slices at the site of infarction on the right and the corresponding area on the left (Figure 1). Other ROIs were selected in both hemispheres in the centrum semiovale, the cerebral peduncle, the thalamus and the knee of the internal capsule. For the corpus callosum, the ROIs were placed in the sagittal median line slice in the genu, the truncus, and the splenium. 3D rotation was used to assure correct localization of the ROIs while analyzing the data along with image overlay technique with anatomical high-resolution images. Examples of the ROI settings are shown in Figure 2. The size of the ROIs ranged from 4 to 16 voxels (voxel size 1.8 * 1.8 * 5 mm^3^), depending on the size of the brain structure. In the small internal capsule, 4 voxels were used. In large areas, i.e., corona radiata and centrum semiovale 16 voxels were used. The same anatomic areas always had equal-sized ROIs over both hemispheres and over all patients. The only exception to this was the case in which the lesion was in the pre-defined ROI and the ROI of the healthy tissue needed to be decreased in size in order to avoid partial volume effects.

### Volume Analysis

**Figure 3:**
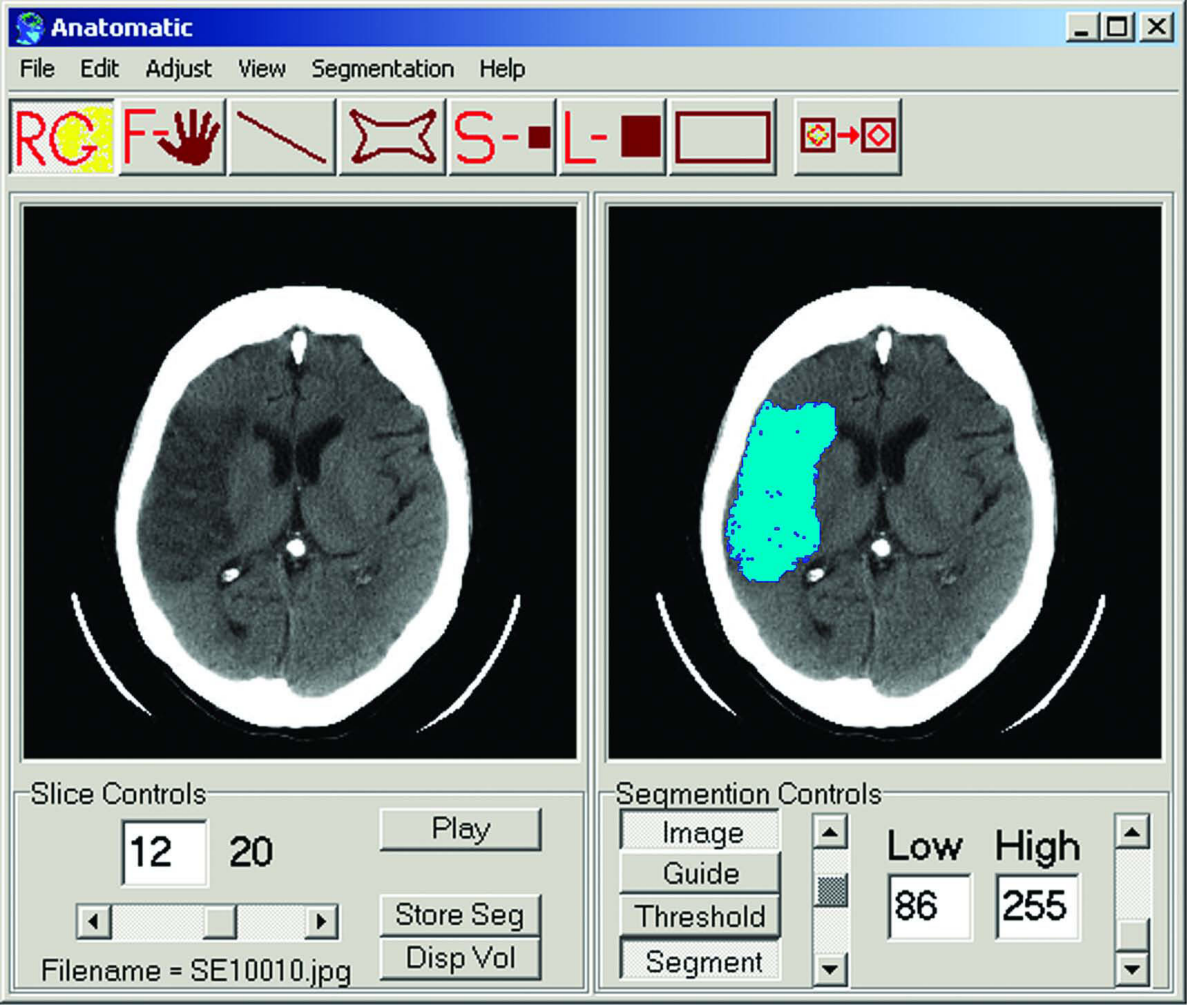
**Software for volumetric analysis**. The user interface of Anatomatic™ 2.23 software was used in the volumetric analysis. The figure on the left represents the original axial CT slice depicting a large temporal lobe infarction. The figure on the right represents the accurately segmented area of infarction.

The volumetric analyses were carried out from the acute-stage CT and the chronic-stage MRI. The calibrated semi-automatic segmentation software Anatomatic™ 2.23[16] was used to measure the ischemic lesion volumes on the CT and FLAIR sequences (Figure 3). All the images were analysed by the same reader (S.M.) who was blinded to the clinical data. The intra-rater variability in volume measurements was tested with ten randomly chosen patients, who were also used to study inter-rater variability. To avoid possible systematic errors of the FLAIR image due to a large slice thickness and slice gap, a 3D *T *_2_-weighted image (voxel size 0.84*0.84*0.9 mm^3^) from the same session was segmented, calculated and tested against the FLAIR lesion volume. All analyses were supervised by one senior experienced neuroradiologist (P.D.).

### Statistical Analysis

As most of the data was heavily skewed, we used only nonparametric tests. The Wilcoxon signed-rank test was used to assess the difference between the acute and chronic volumes and the neurological test scores, and the MD and FA values between the right and left hemispheres. The Mann-Whitney U test was used to determine the effect of thrombolytic therapy and the location of the lesion, i.e., MCA vs. PCA. Spearman's correlation was used to test the relationships between the volume and the DTI parameters, and between the volume and the neurological scores. It was also used to test for a relationship between the change in neurological tests (chronic test - acute test) and the change in lesion volume (chronic volume - acute volume). Bland-Altman plots were used to evaluate the intra- and inter-rater reliability [17]. In the Bland-Altman plot, the difference between two measurements is plotted against their mean. In order to prove good agreement, 95% of those points should fall within the limits of agreement (LA), calculated as LA = mean ± 2 SD [17]. All the analyses were performed on SPSS 16.0 for Windows. Initially, p-values less than 0.05 were considered statistically significant. Then the Bonferroni post-hoc test was used to correct the familywise error rate in the analyses of the numerous ROIs.

## Results

Eight of the initial 41 patients were excluded from the study. Of those excluded, seven patients had no signs of an infarction on chronic-phase MRI, and one patient had only microangiopathic changes. The patient characteristics and lesion locations of the remaining 33 patients are reported in Table 1. The patients included 26 males (79%) and 7 females (21%). The mean age of the study subjects was 65.9 ± 8.7 years. Ten (30%) of the patients received thrombolytic therapy. Most of the patients had their occlusion in the MCA. Seven patients did not show any signs of an infarction on the acute-phase CT but they had a visible lesion in the chronic-phase MRI examination and were thus included in the study.

**Table 1:** Patient characteristics and lesion volumes

Sex	Age	TT	Location	Acute volume (mL)	Chronic volume (mL)
M	57	No	MCA, lower dorsal temporal lobe	0.00	2.48
M	73	No	MCA and ACA, peri callosum	2.92	21.45
M	66	No	MCA, dorsal frontal lobe and temporal lobe	7.71	70.46
F	57	No	MCA, upper temporal lobe	0.00	9.44
M	75	No	PCA, temporal occipital lobe	2.98	26.46
M	64	No	MCA, corona radiata	0.26	1.88
M	59	Yes	MCA, corona radiata	0.05	0.82
M	71	No	MCA, corona radiata, capsula externa	1.25	8.84
M	56	No	PCA, temporal occipital lobe, thalamus, corona radiata	2.19	51.12
M	46	No	MCA, corona radiata	0.35	1.34
M	59	Yes	PCA, occipital lobe	0.69	3.73
M	73	No	MCA, nucleus lentiformis	0.23	4.5
M	69	No	MCA, temporal lobe	0.33	84.96
M	55	Yes	PCA, occipital lobe	3.46	10.9
M	62	No	MCA, dorsal temporal lobe	1.87	27.31
M	70	Yes	MCA, corona radiata, capsula externa	0.09	0.82
M	64	No	MCA, temporal lobe	2.36	80.7
M	55	Yes	MCA, upper temporal lobe	1.49	15.99
F	77	Yes	MCA, corona radiata	0.02	0.54
M	66	No	Watershed, frontal lobe, frontoparietal border	0.25	2.85
M	78	No	Watershed, dorsal frontal lobe and parietal lobe	0.00	2.71
F	62	No	MCA, temporal lobe	0.00	0.57
F	62	Yes	MCA, corona radiata, capsula externa	0.22	1.18
M	73	No	MCA, corona radiata	0.48	1.67
M	62	No	MCA, corona radiata, capsula interna	0.36	0.89
M	78	No	MCA, corona radiata	0.35	0.57
F	79	No	MCA, temporal lobe	13.55	102.96
M	72	Yes	MCA, upper dorsal temporal lobe	4.05	37.06
F	75	No	MCA, corona radiata, capsula interna	0.00	0.76
M	52	No	MCA, corona radiata, capsula interna, nucleus caudatus, nucleus lentiformis	0.00	1.56
F	73	Yes	MCA, temporal lobe	2.15	14.06
M	75	Yes	PCA, cortical infarction	0.00	0.55
M	58	No	MCA, corona radiata, putamen, capsula externa; PCA, deep white matter	8.5	15.34

TT, Thrombolytic Therapy; MCA, Middle Cerebral Artery; ACA, Anterior Cerebral Artery; PCA, Posterior Cerebral Artery.

### DTI results

**Figure 4:**
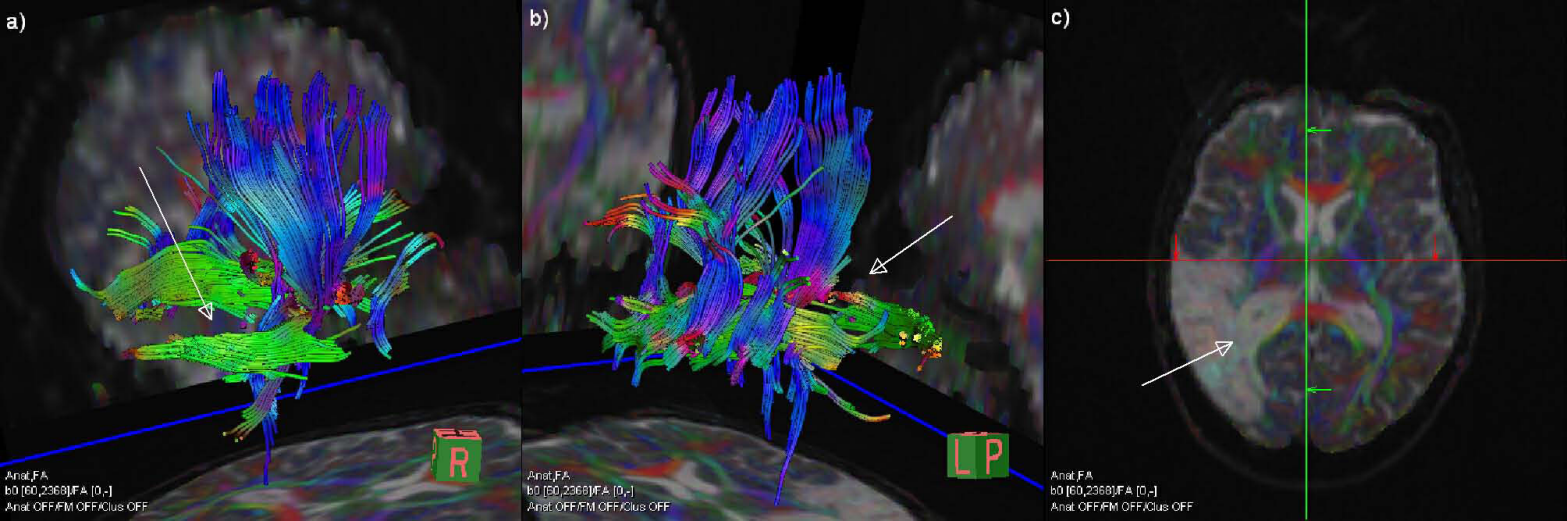
**Tractography in the lesion area**. Example tractography images of trackts completely destroyed after infarction in two different angles (a, b) and the corresponding ADC map in a 64-year-old male patient. The missing fiber trackts are indicated with arrows.

In patients with chronic infarctions all possible tracts in the area of lesion disappeared as shown in Figure 4. In addition, in patients with visible Wallerian degeneration the fiber tracts in the area of mesencephalon were distorted. No dependency between location of lesion and regional changes of FA or MD was seen. Both FA and MD changes were seen in any location that had suffered from the ischemic or infarction changes and associated Wallerian degeneration.

The FA values in the right hemisphere were lower than those in the left hemisphere, except in the thalamus that showed no difference. The MD values in the right hemisphere were higher than those in the left hemisphere. The descriptive values of the FA and MD values in both hemispheres are represented in Table 2. The difference between the hemispheres was significant in all comparisons except the FA in the thalamus. The results also survived the Bonferroni correction. The FA values [mean (SD) median] of the corpus callosum were [0.80 (0.06) 0.79] in the rostrum, [0.63 (0.12) 0.66] in the truncus and [0.84 (0.07) 0.84] in the splenium. The respective MD (× 10^-3^mm^2^s^-1^) values of the corpus callosum were [0.77 (0.09) 0.75] in the rostrum, [0.95 (0.18) 0.92] in the truncus and [0.74 (0.10) 0.73] in the splenium. Thrombolytic therapy had no effect on the DTI results. There was no difference in the DTI values of the MCA and PCA territories, and no correlation with age was found.

**Table 2:** DTI data

	Infarct side (right side)		Unaffected side (left side)		
	Mean (SD)	Median	Mean (SD)	Median	p-value*
FA					
Infarct/undamaged contralateral area	0.17 (0.09)	0.16	0.42 (0.18)	0.43	< 0.001
Internal capsule genu	0.69 (0.11)	0.70	0.72 (0.07)	0.73	0.014
Thalamus	0.33 (0.06)	0.32	0.32 (0.04)	0.32	0.381
Cerebral peduncle	0.75 (0.08)	0.75	0.79 (0.06)	0.79	0.003
Centrum semiovale	0.56 (0.09)	0.58	0.60 (0.09)	0.62	0.012

**MD**					

Infarct/undamaged contralateral area	1.79 (0.55)	1.63	0.80 (0.14)	0.76	< 0.001
Internal capsule genu	0.72 (0.07)	0.72	0.69 (0.05)	0.70	0.021
Thalamus	0.87 (0.18)	0.82	0.77 (0.06)	0.76	< 0.001
Cerebral peduncle	0.74 (0.07)	0.74	0.70 (0.05)	0.69	0.002
Centrum semiovale	0.72 (0.05)	0.72	0.70 (0.05)	0.69	0.007

Mean values, standard deviations (SD) and medians of fractional anisotropy (FA) and mean diffusivity (MD) (× 10^-3^mm^2^s^-1^) scores in different regions in diffusion tensor imaging analysis.

* The difference between the right and left sides, Wilcoxon signed-rank test.

### Computerised volumetric analysis of infarctions

The lesion volumes are reported in Table 1. The mean lesion volume in the acute phase was 2.24 ± 3.19 mL (median 0.97 mL) and in the chronic phase was 18.37 ± 28 mL (median 3.73 mL). The difference between the acute- and chronic-phase volumes was significant at p < .001. The change in volume ranged from 0.22 to 89.42 mL. The mean change in volume was 16.61 ± 26.11 mL and the median change was 3.04 mL [0.86 to 21.01 (IQR 25% to 75%)]. Although patients who received thrombolytic therapy did have slightly smaller lesion volumes (Table 1), the difference was not statistically significant (p = 0.305). In addition, we found no significant difference between the MCA and PCA infarction volumes in either the acute (p = 0.358) or chronic phase (p = 0.658). There was also no correlation between patient age and either acute (p = 0.937) or chronic (p = 0.980) lesion volume.

The intra-rater variability for the acute CT volume analyses was 0.04 ± 0.05 mL and for the chronic FLAIR volume analyses it was 0.17 ± 0.19 mL. All measured intra-rater volumes were within the boundaries of two SDs in the Bland-Altman plot. The inter-rater variability for the CT and FLAIR volume analyses were 0.15 ± 0.17 mL and 1.16 ± 1.36 mL, respectively. The Bland-Altman plot for the inter-rater CT volume data had 87% of data points within the boundary limits. For the FLAIR volume data, 90% of the data points for the inter-rater variation analysis were within the boundary limits in the Bland-Altman plot. The FLAIR lesion volume showed excellent correlation (r^2 ^= 0.995) with the 3D lesion volume (data not shown).

### Correlation between lesion volume and DTI results

**Figure 5:**
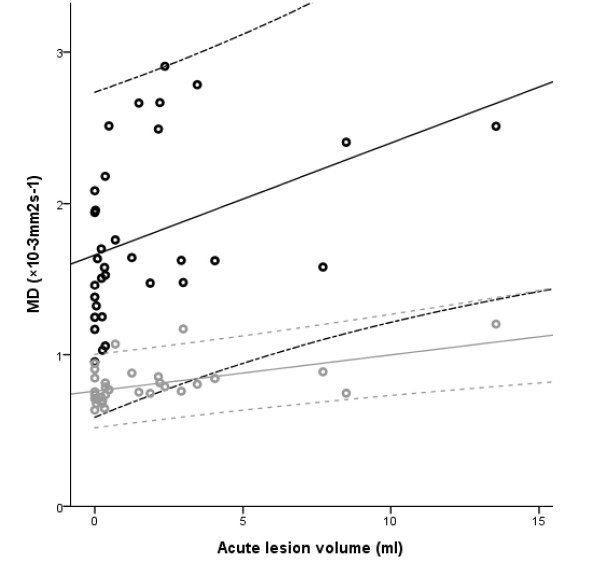
**Association between acute lesion volume and MD**. Correlation between acute-stage lesion volume and mean diffusivity within the lesion (black) and the corresponding contralateral area (grey) with 95% confidence intervals.

Correlations between the chronic FA value and the lesion volume in the acute stage were found in the right internal capsule and the centrum semiovale. The chronic lesion volume correlated with FA only within the lesion. The MD values in several right hemispheric areas correlated with the lesion volumes in both the acute and chronic stages. Correlation between acute lesion volume and MD in both the lesion and the corresponding contralateral area is presented in Figure 5. There was no inter-correlation between the two MDs in the figure (p = 0.395). In the corresponding contralateral area, a correlation was found between the acute volume and the chronic MD value, and between the chronic volume and FA value. The chronic MD in corpus callosum splenium correlated with the acute lesion volume. All the correlations are reported in Table 3. A few weak correlations in the measured volumes were found in the DTI results of the contralateral hemisphere.

**Table 3:** Correlation between volume and DTI

	Acute lesion volume		Chronic lesion volume	
	FA	MD	FA	MD
Corpus callosum rostrum	-0.179 (0.318)	0.236 (0.185)	-0.297 (0.093)	0.264 (0.138)
Corpus callosum truncus	-0.173 (0.337)	0.190 (0.290)	-0.290 (0.101)	0.262 (0.141)
Corpus callosum splenium	-0.138 (0.444)	**0.386 (0.027)**	0.053 (0.770)	0.227 (0.204)

**Right hemisphere**				

Infarct	-0.274 (0.123)	**0.498 (0.003)**	**-0.406 (0.019)**	0.352 (0.045)
Cerebral peduncle	-0.139 (0.439)	**0.463 (0.007)**	-0.194 (0.281)	**0.573 (0.000)**
Thalamus	-0.327 (0.063)	0.361 (0.039)	-0.307 (0.083)	0.349 (0.047)
Internal capsule genu	**-0.522 (0.002)**	**0.566 (0.001)**	-0.291 (0.100)	**0.511 (0.002)**
Centrum semiovale	**-0.522 (0.002)**	**0.413 (0.017)**	-0.197 (0.272)	**0.396 (0.022)**

**Left hemisphere**				

Corresponding area of infarction	-0.129 (0.474)	**0.435 (0.011)**	**-0.386 (0.026)**	0.274 (0.122)
Cerebral peduncle	0.059 (0.745)	0.361 (0.039)	0.091 (0.614)	0.184 (0.305)
Thalamus	-0.376 (0.031)	0.176 (0.327)	-0.298 (0.092)	0.045 (0.803)
Internal capsule genu	**-0.421 (0.015)**	0.351 (0.045)	-0.131 (0.468)	0.168 (0.351)
Centrum semiovale	0.030 (0.870)	0.001 (0.997)	0.154 (0.391)	-0.041 (0.819)

Spearman's correlation between the infarct volume and the fractional anisotropy (FA) and mean diffusivity (MD) (× 10^-3^mm^2^s^-1^) values in different regions.

Numbers in parentheses are the p-values. Statistically significant results that survived the Bonferroni correction are highlighted in bold.

### Neurological findings

The patients' neurological deficits improved over the study period. The acute stage neurological scores [median (range)] for the NIHSS, BI, RS and MMSE were [3 (0 to 10)], [87.5 (30 to 100)], [2 (0 to 5)], and [27 (21 to 31)], respectively. The chronic stage scores were [0 (0 to 6)] for the NIHSS, [100 (90 to 100)] for the BI, [1 (0 to 3)] for the RS, and [28 (22 to 30)] for the MMSE. The differences between the acute and chronic test scores were significant at a *p *value <.001 in all tests except the MMSE that showed no difference.

### Correlation between clinical scores and lesion volume

**Figure 6:**
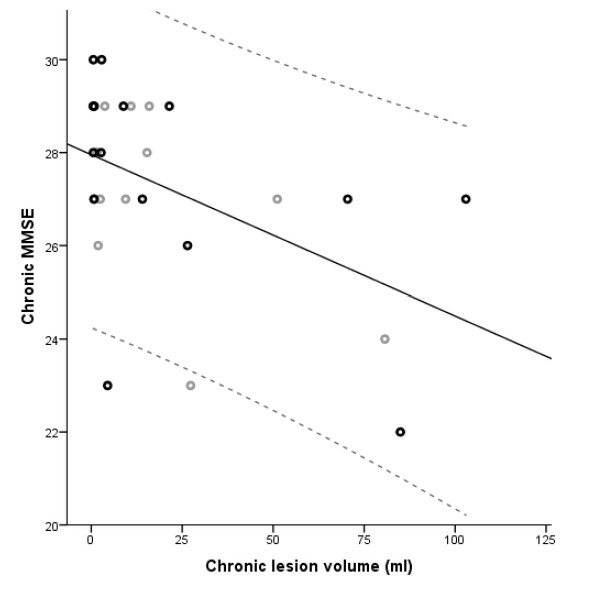
**Correlation between chronic lesion volume and MMSE**. Correlation between chronic-stage lesion volume and mini-mental state examination (MMSE) with 95% confidence interval. Patients are divided into older (black) and younger (grey) than 65 years.

A statistically significant negative correlation between the neurological test scores and the lesion volumes was found only between the chronic MMSE score and the lesion volume in the chronic stage (*r *= -0.443 at *p *= 0.015) (Figure 6). No effect of age was found. The change in the NIHSS score also tended to correlate negatively with the change in the volume (*r *= -0.393 at *p *= 0.039), but did not survive the Bonferroni correction.

## Discussion

Both DTI and the estimation of lesion volumes have been useful in assessing the clinical state of ischemic stroke patients. Therefore, the degree of change between the volumes in the acute and chronic stages was shown here to be associated with the degree of microscopic tissue modifications detected with DTI. Indeed, in the 33 right-sided infarct patients included in this study, our results showed promise in combining the two analyses. Furthermore, we found a prognostic value of the acute-stage volume.

The FA and MD values reported in previous studies [8,18,19] are similar to those we recorded. The elevation in the MD value of the ipsilateral thalamus without a change in the FA value has been previously reported by Hervé *et al*. (2005) [20]. In the study by Liang *et al. *[6], there were no differences between the FA and MD values of contralateral hemispheres in either the study patients or those in the control group. These results suggest that the contralateral hemisphere is a reliable point of comparison. Despite the lack of lateralization in a healthy human brain, in these infarct patients the MD value was elevated, and except for the thalamus, the FA value decreased in the right hemisphere in comparison to the contralateral hemisphere. Although there are generally no differences between hemispheres [6,20], specific brain areas, such as those related to speech, are normally lateralized [21] and hence, DTI differences between hemispheres may partly be influenced by normal physiology, not pathology.

Both PCA and MCA lesions were included in this study due to the suspicion of attention deficit. There was no difference between patients with PCA or MCA lesions in either the DTI results or the lesion volumes in these patients. Neglect was an important question in the initial planning of this project and the detailed results will be described elsewhere by neuropsychologists.

In previous reports, our segmentation method has been shown to have good reproducibility with an inter-rater variability of 7% or less and an intra-rater variability of 4% or less [16,20]. The slice thickness used in our current study was relatively large for volume rendering, but the excellent intra-rater variability in this study in both CT and MRI, along with excellent agreement between the FLAIR and 3D-rendered volumes, justifies the validity of the volumetric measurements. The median lesion volumes in the acute and chronic phases were 0.97 and 3.73 mL, respectively. These are considerably smaller in comparison with previous studies done with similar patient groups [22]. Although MRI is known to be superior to CT [12,13] and lesions not seen on CT may be detected on MRI, our initial experience has been positive regarding the CT diagnosis, including conventional CT, angiography, and perfusion, in the hyperacute stage.

We found correlations between the lesion volumes and MD. In several ROIs, including the lesion area, the MD values correlated with lesion volumes in both the acute and chronic stage. This means that the lesion volume in the acute stage predicts the diffusion elevation in the chronic stage. This connection is also seen with the chronic lesion volume and the MD values. Alterations in MD may derive from alterations in both decreased longitudinal and increased radial diffusion, indicating axonal and myelin damage, respectively [23]. Therefore, the evaluation of pure MD is somewhat limited and in the future should be accompanied by the evaluation of both longitudinal (λ_1_) and radial $\sqrt{\left(\lambda_{2}^{2}+\lambda_{3}^{2}\right)}$ diffusivities, along with fiber tracking method to assure the direction of λ_1_. The analysis may be further improved by including the calculation of crossing fibers [24-26] both before and after the infarction.

The negative correlation between the chronic lesion volume and the FA in the infarct area may be caused by more thorough tissue destruction in larger lesions, leading to smaller anisotropy. However, this interpretation is complicated if lesions are located in regions with crossing fibers. With only one fiber direction degenerating, increased FA may occur. Lower FA in the internal capsule and the centrum semiovale was predicted by the acute lesion volumes. Larger measurable lesions in the acute phase may lead to more extensive disintegration of the ipsilateral white matter tracts. This is manifested in the chronic phase as decreased FA values of the internal capsule and centrum semiovale.

A simultaneous decrease in the size and deterioration of the DTI results of the corpus callosum has been reported over time after large MCA strokes [27]. We found tendency to correlation between the acute lesion volume and the MD values of the corpus callosum splenium. The weak correlations with the left hemisphere cannot be explained in our study. The correlation between the acute lesion size and the chronic MD value in the left cerebral peduncle and the internal capsule might indicate that a larger volume in the acute stage tends to predict white matter tract disintegration in the contralateral hemisphere. The correlation between the FA values of the internal capsules and the acute lesion volumes supports this interpretation. However, there were no similar relations with the chronic lesion volumes to support this theory.

To improve the accuracy of the results, we will be increasing the number of gradient encoding directions and applying diffusion spectrum imaging (DSI) in our future studies. First, the signal to noise ratio and the accuracy of DTI results improve when increasing the number of gradient encoding directions. [28] In this study, 12 directions were acquired that we have since increased to 20 directions. Too few sampling directions lead to larger variations, biases and correlations between tensor orientations and diffusion characteristics, especially at high FA. [28] These effects, especially in the lesion area, had little effect in this study due to the relatively low FA except for the cerebral peduncle. Second, methods to correct for the effect of crossing fibers have been recently developed to solve the problem [25,26] that possibly limits the interpretation of the results of the current study. In our patient cohort, the complete disappearance of all fiber tracts within the lesion area contradicts this limitation. However, in healthy brain tissue DSI is needed for further evaluation of regional differences as yet hidden by DTI.

The lesion volume has shown significant correlation with the NIHSS and BI [9,10,22] scores. Nevertheless, the only correlation between the lesion volume and neurological test results we found was between the chronic volume and the chronic MMSE score. In our study, there was a narrower range between the baseline and chronic scores than in the reference studies. Compared with the previous studies, our patients showed considerably better results in neurological tests in both the acute and chronic stages. The lesion volumes were also smaller in our study than in the reference studies. In addition to these differences, the volumes attained in previous studies were measured slightly differently. Baird *et al. *[10] used MR perfusion and DWI in their study. They reported a strong correlation between the change in volume and the change in NIHSS scores. In their study, the acute lesion volume was defined as all tissue at risk and was the larger of the volumes at the acute time point. The chronic lesion volume was measured using DWI. However, when the authors measured both volumes with DWI only, no correlation was found between the change in the volume and the change in the NIHSS score. We found modest correlations between the change in volume and the change in the NIHSS score. As acute lesions with symptom onset of less than 3-4 h may not be detectable on CT - which was the case in seven patients - our results may be slightly affected. However, our results concord with previous studies[10]. Perhaps the lack of correlations to other neurological tests partly derives from the acute-stage CT instead of MRI. The side of the infarction may also be an influential factor. Fink *et al. *[29] reported that patients with the same NIHSS score had greater lesion volumes if the lesion was on the right side rather than on the left side.

The fact that the chronic neurological tests and the MRI could not practically be done in parallel may have caused bias. However, Gaudinski *et al. *[30] reported that the final infarct volume can be predicted from the lesion size determined by imaging thirty days after the onset of symptoms, and that there was only a 5% decrease in the infarct volume between 30 and 90 days. The volume difference was in the range of inter-reader variability. Based on the results of Gaudinski *et al. *[30], the chronic lesion volumes we measured can be viewed as accurate representations of the lesion size at six months after infarction. This enables us to correlate the lesion volumes with neurological test scores. Similar assumptions regarding the FA and MD values could not be made. FA decreases at least 12 weeks [6] and MD increases at least six months [20] or longer after infarction. MD is closely related to DWI signal intensity that has been shown to decrease after the first week [31]. Also, apparent diffusion coefficient in DWI has been shown to develop long after the acute phase, increasing to subnormal after an initial decrease [31,32]. With information lacking at 6-12 months after infarction, we could not correlate FA and MD values to clinical scores.

## Conclusions

Based on our findings, in which significant correlations were found between infarct volumes and pathological DTI values, we believe that DTI analysis can prove to be a good supplementary technique in analysing the damage to brain tissue during the chronic phase of stroke recovery.

## Competing interests

The authors declare that they have no competing interests.

## Authors' contributions

MR assisted in the technical aspects of the volumetric analyses and wrote the final manuscript. EJ performed the DTI analysis and significantly contributed to drafting the manuscript. SM performed the volumetric analyses. PD supervised both the volumetric and DTI analyses and significantly assisted in writing the manuscript. JO performed the neurological testing and, together with SS, conceived of the study and participated in its design and coordination. All authors read and approved the final manuscript.

## Pre-publication history

The pre-publication history for this paper can be accessed here:

http://www.biomedcentral.com/1471-2342/10/21/prepub
